# Supplementary material for: Wholegrain Consumption and Risk Factors for Cardiorenal Metabolic Diseases in Chile: A Cross-Sectional Analysis of 2016–2017 Health National Survey
Source: Nutrients. 2020 Sep 14;12(9):2815. doi: 10.3390/nu12092815 (PMC7576471; doi:10.3390/nu12092815)
Supplement: Supplementary file 1 [file nutrients-12-02815-s001.pdf]

Supplementary Table 1. Association between WG consumption and cardiorenal metabolic outcomes.

Outcomes	Non-consumer (n:6,369,362)*	Sporadic WG consumer (n: 2,316,451)*	Regular WG consumer (n: 3,124,834)*
**MetS**			
Model 1	Ref.	1.06 (0.77-1.48)	0.82 (0.59-1.16)
Model 2	Ref.	1.12 (0.77-1.63)	0.94 (0.63-1.39)
Model 3	Ref.	1.11 (0.76-1.62)	0.89 (0.60-1.30)
**High WC**			
Model 1	Ref.	1.00 (0.67-1.50)	0.80 (0.56-1.16)
Model 2	Ref.	0.83 (0.46-1.47)	0.75 (0.46-1.23)
Model 3	Ref.	0.79 (0.45-1.39)	0.68 (0.41-1.12)
**Low HDL**			
Model 1	Ref.	0.87 (0.61-1.25)	0.82 (0.58-1.15)
Model 2	Ref.	0.87 (0.61-1.25)	0.82 (0.58-1.15)
Model 3	Ref.	0.88 (0.62-1.25)	0.83 (0.59-1.16)
**HTG**			
Model 1	Ref.	0.83 (0.58-1.20)	0.78 (0.54-1.12)
Model 2	Ref.	0.86 (0.60-1.25)	0.96 (0.65-1.42)
Model 3	Ref.	0.85 (0.59-1.23)	0.93 (0.63-1.36)
**High BP**			
Model 1	Ref.	1.04 (0.73-1.49)	0.64 (0.45-0.92)
Model 2	Ref.	1.06 (0.74-1.54)	0.67 (0.45-0.99)
Model 3	Ref.	1.03 (0.71-1.50)	0.61 (0.41-0.91)
**IFG**			
Model 1	Ref.	1.21 (0.86-1.71)	0.95 (0.66-1.36)
Model 2	Ref.	1.25 (0.89-1.76)	1.00 (0.67-1.49)
Model 3	Ref.	1.26 (0.90-1.77)	1.00 (0.68-1.47)
**CKD**			
Model 1	Ref.	1.26 (0.58-2.74)	0.47 (0.22-0.99)
Model 2	Ref.	1.33 (0.63-2.82)	0.47 (0.21-1.04)
Model 3	Ref.	1.37 (0.66-2.86)	0.49 (0.22-1.09)
**Albuminuria**			
**Model 1**	Ref.	1.48 (0.90-2.43)	1.41 (0.81-2.46)
**Model 2**	Ref.	1.53 (0.97-2.42)	1.48 (0.87-2.52)
**Model 3**	Ref.	1.53 (0.97-2.43)	1.43 (0.84-2.43)

MetS, metabolic syndrome; WC, waist circumference; HDL, high density lipoprotein; HTG, hypertriglyceridemia; BP, blood pressure; IFG, impaired fasting glucose; CKD, chronic kidney disease; Ref. reference. Model 1 included geographical area (urban *vs.* rural), age (18-24, 25-44, 45-64, >65 years), sex. Model 2 was additionally adjusted for BMI (<25, 25-30, >30 kg/m^2), education level (<8, 8-12, >13 years of education), tobacco use (current smokers *vs.* former and non-smokers), frequency of alcohol consumption (never, ≤1 time/month, 2-4 times/month, 2-3 times/week, >3 times/week) and quantity of alcohol glasses typically consumed (continuous, 0 for non-drinkers), physical activity (GPAQ, low, moderate, high), diabetes (self-reported or fasting blood glucose >126 mg/dl) and previous cardiovascular disease (self-reported of acute myocardial infarction, stroke or peripheral artery disease). For renal outcomes (CKD and albuminuria) the model also included hypertension (self-reported, medication use or systolic/diastolic BP >140/90mmHg). Model 3 was further adjusted for consumption of fruits, vegetables (times per week) and legumes (categorical, 3 levels). Albuminuria was available in 2,432 interviewed participants representing a population of 8,385,402 and ordinal regression instead of logistic regression was used. *n after applying sampling weights

Supplementary Table 2. Sensitivity analysis of the association between WG consumption and cardiorenal metabolic outcomes.

	**Non-consumer**	**Sporadic WG consumer**	**Regular WG consumer**
**Urban population (n:10,504,996)**	(n: 5,478,333)	(n: 2,086,804)	(n: 2,939,857)
MetS (n:4,343,671)	Ref.	1.08 (0.71-1.64)	0.92 (0.60-1.41)
High WC (n:8,065,011)	Ref.	0.91 (0.49-1.68)	0.75 (0.44-1.27)
Low HDL (n:5,442,976)	Ref.	0.85 (0.58-1.25)	0.90 (0.62-1.29)
HTG (n:3,613,556)	Ref.	0.81 (0.55-1.21)	0.97 (0.64-1.46)
High BP (n:3,880,264)	Ref.	1.028 (0.68-1.55)	0.55 (0.35-0.85)
IFG (n: 2,625,448)	Ref.	1.22 (0.84-1.75)	1.04 (0.69-1.57)
CKD (n:292,438)	Ref.	1.44 (0.63-3.30)	0.53 (0.22-1.29)
Albuminuria (n: 691,948 / 7,495,138)	Ref.	1.58 (0.95-2.64)	1.45 (0.80-2.64)
**BMI>18.5kg/m^2 (11,702,991)**	(n: 6,315,847)	(n: 2,281,780)	(n: 3,105,363)
MetS (n:4,919,902)	Ref.	1.11 (0.76-1.62)	0.88 (0.60-1.30)
High WC (n:9,040,161)	Ref.	0.79 (0.45-1.39)	0.66 (0.40-1.09)
Low HDL (n:6,154,385)	Ref.	0.89 (0.63-1.27)	0.82 (0.58-1.15)
HTG (n:4,110,102)	Ref.	0.86 (0.60-1.24)	0.93 (0.63-1.36)
High BP (n:4,426,749)	Ref.	1.05 (0.72-1.53)	0.61 (0.41-0.92)
IFG (n: 2,917,786)	Ref.	1.25 (0.89-1.76)	1.01 (0.68-1.48)
CKD (n:325,250)	Ref.	1.46 (0.69-3.09)	0.56 (0.25-1.22)
Albuminuria (n:796,102 / 8,338,154)	Ref.	1.57 (1.00-2.46)	1.54 (0.91-2.58)
**Excluding alcohol dependence (n:11,799,877)**	(n: 6,361,090)	(n: 2,313,952)	(n: 3,124,834)
MetS (n:4,923,256)	Ref.	1.11 (0.75-1.62)	0.88 (0.60-1.30)
High WC (n:9,036,751)	Ref.	0.78 (0.44-1.37)	0.68 (0.41-1.12)
Low HDL (n:6,156,467)	Ref.	0.88 (0.61-1.25)	0.83 (0.59-1.16)
HTG (n:4,112,544)	Ref.	0.85 (0.59-1.22)	0.92 (0.63-1.35)
High BP (n:4,446,048)	Ref.	1.05 (0.72-1.53)	0.61 (0.41-0.92)
IFG (n: 2,937,190)	Ref.	1.24 (0.88-1.74)	1.00 (0.68-1.47)
CKD (n:343,866)	Ref.	1.37 (0.65-2.86)	0.49 (0.22-1.09)
Albuminuria (n:813,188 / 8,380,208)	Ref.	1.53 (0.96-2.42)	1.43 (0.84-2.44)
**Free of diabetes (n:10,297,606)**	(n: 5,537,343)	(n: 2,029,862)	(n: 2,730,400)
MetS (n:3,766,513)	Ref.	1.15 (0.78-1.70)	0.88 (0.60-1.30)
High WC (n:7,646,014)	Ref.	0.82 (0.46-1.45)	0.66 (0.39-1.12)
Low HDL (n:5,227,893)	Ref.	0.92 (0.63-1.35)	0.82 (0.57-1.19)
HTG (n:3,382,378)	Ref.	0.89 (0.60-1.31)	0.92 (0.63-1.35)
High BP (n:3,498,030)	Ref.	0.93 (0.61-1.41)	0.56 (0.36-0.88)
IFG (n: 1,428,813)	Ref.	1.34 (0.86-2.10)	0.79 (0.47-1.35)
CKD (n:241,652)	Ref.	1.59 (0.72-3.53)	0.62 (0.25-1.53)
Albuminuria (n:498,717 / 7,053,847)	Ref.	1.38 (0.78-2.42)	0.82 (0.47-1.40)
**Free of CVD (n:10,672,878)**	(n: 5,662,813)	(n: 2,099,118)	(n: 2,910,947)
MetS (n:4,242,555)	Ref.	1.08 (0.72-1.63)	0.92 (0.61-1.39)
High WC (n:8,047,451)	Ref.	0.94 (0.53-1.68)	0.73 (0.44-1.23)

Low HDL (n:5,575,037)	Ref.	0.84 (0.57-1.24)	0.76 (0.53-1.09)
HTG (n:3,721,997)	Ref.	0.85 (0.58-1.25)	0.94 (0.62-1.41)
High BP (n:3,702,663)	Ref.	1.07 (0.72-1.58)	0.64 (0.41-0.98)
IFG (n: 2,393,683)	Ref.	1.25 (0.86-1.80)	0.96 (0.63-1.46)
CKD (n:194,287)	Ref.	0.89 (0.34-2.34)	0.62 (0.26-1.47)
Albuminuria (n:635,400 / 7,444,201)	Ref.	1.26 (0.75-2.12)	1.32 (0.72-2.40)

*The numbers in brackets are the participants positive for that particular condition. †Odds ratio are shown in the fully adjusted model. MetS, metabolic syndrome; WC, waist circumference; HDL, high density lipoprotein; HTG, hypertriglyceridemia; BP, blood pressure; IFG, impaired fasting glucose; CKD, chronic kidney disease. Model included geographical area (urban vs. rural), age (18-24, 25-44, 45-64, >65 years), sex, body mass index (BMI, <25, 25-30, >30 kg/m^2), education level (<8, 8-12, >13 years of education), tobacco use (current smokers *vs.* non-smokers and former smokers), frequency of alcohol consumption (never, ≤1 time/month, 2-4 times/month, 2-3 times/week, >3 times/week) and quantity of alcohol glasses typically consumed (continuous, 0 for non-drinkers), physical activity using the Global Physical Activity Questionnaire (GPAQ, low, moderate, high), hypertension (self-reported, medication use or systolic/diastolic BP > 140/90 mmHg), diabetes (self-reported or fasting blood glucose >126 mg/dl) and previous cardiovascular disease (self-reported of acute myocardial infarction, stroke or peripheral artery disease) and consumption of fruits, vegetables (times per week) and legumes (categorical, 3 levels). For renal outcomes (CKD and albuminuria) the model also included hypertension (self-reported, medication use or systolic/diastolic BP >140/90mmHg). Ordinal regression instead of logistic regression was used for albuminuria. *n after applying sampling weights
